# Characterization of Genome-Methylome Interactions in 22 Nuclear Pedigrees

**DOI:** 10.1371/journal.pone.0099313

**Published:** 2014-07-14

**Authors:** Nongluk Plongthongkum, Kristel R. van Eijk, Simone de Jong, Tina Wang, Jae Hoon Sul, Marco P. M. Boks, René S. Kahn, Ho-Lim Fung, Roel A. Ophoff, Kun Zhang

**Affiliations:** 1 Department of Bioengineering, University of California San Diego, La Jolla, California, United States of America; 2 Center for Neurobehavioral Genetics, Semel Institute for Neuroscience and Behavior, David Geffen School of Medicine at the University of California Los Angeles, Los Angeles, California, United States of America; 3 Department of Psychiatry, Rudolf Magnus Institute of Neuroscience, University Medical Center Utrecht, Utrecht, The Netherlands; 4 Department of Computer Science, University of California Los Angeles, Los Angeles, California, United States of America; Universität des Saarlandes, Germany

## Abstract

Genetic polymorphisms can shape the global landscape of DNA methylation, by either changing substrates for DNA methyltransferases or altering the DNA binding affinity of *cis*-regulatory proteins. The interactions between CpG methylation and genetic polymorphisms have been previously investigated by methylation quantitative trait loci (mQTL) and allele-specific methylation (ASM) analysis. However, it remains unclear whether these approaches can effectively and comprehensively identify all genetic variants that contribute to the inter-individual variation of DNA methylation levels. Here we used three independent approaches to systematically investigate the influence of genetic polymorphisms on variability in DNA methylation by characterizing the methylation state of 96 whole blood samples in 52 parent-child trios from 22 nuclear pedigrees. We performed targeted bisulfite sequencing with padlock probes to quantify the absolute DNA methylation levels at a set of 411,800 CpG sites in the human genome. With mid-parent offspring analysis (MPO), we identified 10,593 CpG sites that exhibited heritable methylation patterns, among which 70.1% were SNPs directly present in methylated CpG dinucleotides. We determined the mQTL analysis identified 49.9% of heritable CpG sites for which regulation occurred in a distal *cis*-regulatory manner, and that ASM analysis was only able to identify 5%. Finally, we identified hundreds of clusters in the human genome for which the degree of variation of CpG methylation, as opposed to whether or not CpG sites were methylated, was associated with genetic polymorphisms, supporting a recent hypothesis on the genetic influence of phenotypic plasticity. These results show that *cis*-regulatory SNPs identified by mQTL do not comprise the full extent of heritable CpG methylation, and that ASM appears overall unreliable. Overall, the extent of genome-methylome interactions is well beyond what is detectible with the commonly used mQTL and ASM approaches, and is likely to include effects on plasticity.

## Introduction

DNA methylation represents an important layer of epigenetic regulation on the transcriptional activity of the human genome and plays a crucial role in genomic imprinting, embryonic development and determination of cell type. Accumulating evidence suggests that DNA methylation patterns, rather than being similar within members of the same species, vary from one individual to another [1], [2], [3] due to both genetic and environmental factors [4], [5]. This variability could potentially explain why certain phenotypic outcomes manifest differently across individuals of the same species, including in terms of the susceptibility to and treatability of many human diseases [6], [7].

With the recent advances in DNA methylation assays, a growing number of studies have identified a genetic contribution to inter-individual variation in DNA methylomes. One type of study relies on methylation quantitative trait locus (mQTL) mapping, which identifies genomic polymorphisms associated with variation of CpG methylation in a *cis*-regulatory manner [8], [9], [10], [11]. An alternative approach involves characterizing allele-specific methylation, in which a change in a specific polymorphism leads to the direct loss or gain of DNA methylation [2], [3], [12], [13], [14], [15]. While an increasingly large number of associations between SNPs and CpG sites have been reported in these recent efforts, it remains unclear whether mQTL and ASM analyses are truly uncovering the full extent of genome-methylome interactions. In this study, we performed targeted bisulfite sequencing on human whole blood samples from 96 individuals representing 22 nuclear pedigrees, and took advantage of the parent-child trios using mid-parent offspring (MPO) analysis to fully uncover genome-methylome interactions. We then performed mQTL and ASM analysis on the same samples, and investigated the capability of each method to identify the genetic contribution to inter-sample methylation variability.

## Results

We characterized DNA methylation levels in genomic DNA from the peripheral blood of 96 individuals in 22 nuclear pedigrees of European ancestry, each including one proband with schizophrenia, two unaffected parents and one or two unaffected siblings (a total of 52 trios of two parents and one child). We measured CpG methylation at single base resolution using ∼330,000 bisulfite padlock probes capturing a pre-selected subset of genomic regions, including promoters, enhancers, DNase I hypersensitive sites and other regions known to be variable among different cell types [16]. Note that, like other bisulfite-based methods, 5-methylcytosine and 5-hydroxymethylcytosine are indistinguishable with this assay. In addition, several recent works have shown that variation in cell composition is a confounding factor [17], [18], [19]. In this study, we did not correct for cell composition due to the lack of reference data from pure cell populations, and treated the average methylation of all cells in whole blood as a quantitative trait. On average, we obtained methylation measurements for ∼500,000 CpG sites per sample. A total of 411,800 autosomal CpG sites (and 5,133 on sex chromosomes) had valid methylation measurements in at least 80% of samples. We filtered out CpG sites showing low variability among samples (“static CpG sites”), and focused all further analysis on a subset of 76,408 autosomal variable CpG sites (those with standard deviation of methylation levels across all samples ≥0.1). Hierarchical clustering based on the methylation levels of highly variable autosomal CpG sites (standard deviation ≥0.3) showed a clustering pattern consistent with the family structure (Figure S1 in File S1). While several samples came from individuals with schizophrenia, the sample size here was too small to perform any significant association tests between disease state and either genetic or methylation factors; thus, we focused on treating methylation itself as a quantitative trait and investigating its relation to individual genetic variants.

### MPO identifies CpG sites known to have heritable methylation patterns using trio information

**Figure 1:**
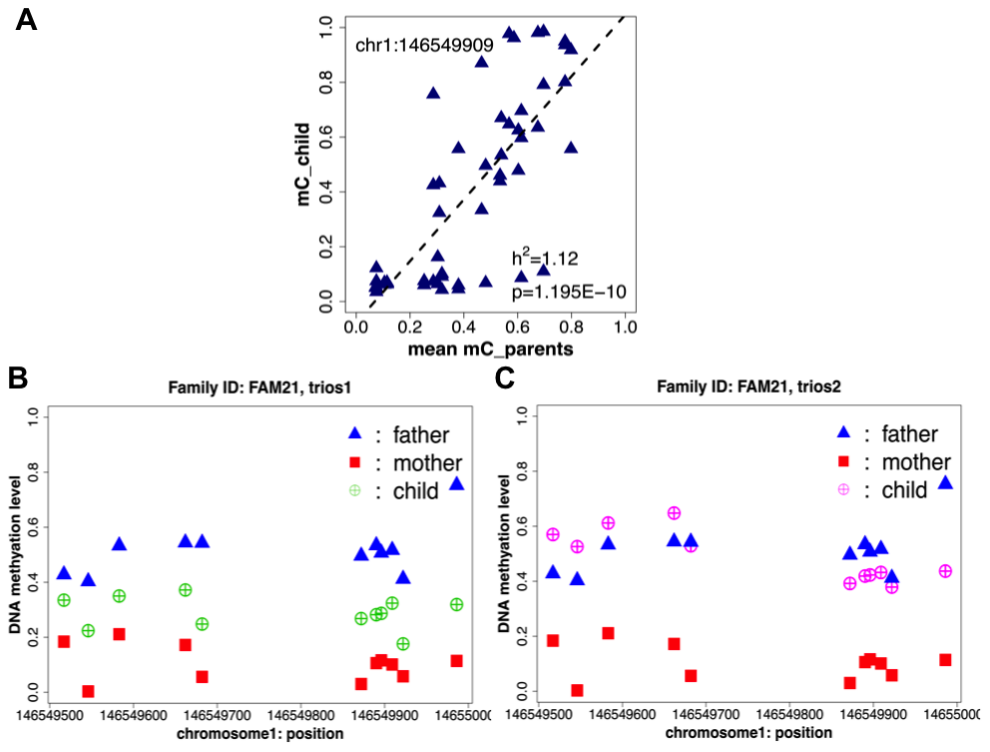
Identification of heritable CpG methylation by mid-parent offspring (MPO) analysis. (a) An example of mid-parent offspring regression of DNA methylation at the CpG site chr1∶146549909. (b,c) DNA methylation level of heritable CpG at chr1∶146549909 and the adjacent heritable CpGs on the same cluster exhibiting consistent pattern of DNA methylation between parents and their offspring on the two trios from the same family.

In order to obtain an independent list of CpG sites where variability in DNA methylation was known to be related to genetic factors, we performed mid-parent offspring (MPO) analysis [20], which analyzes the correlation between the mean methylation level at each CpG site in each parent pair and the methylation level at the same CpG sites in the child (Figure 1a). This family-based analysis of each trio allowed identification of any potential heritable methylation patterns irrespective of the type and frequency of genetic variants (i.e. SNPs, indels, structural genomic variation) or the method of regulation. We identified CpG sites as heritable by requiring a heritability (h^2^) value greater than 0.2 in a minimum of available data in ten trios with a FDR cutoff of 0.05 (with Benjamini-Hochberg correction).

**Figure 2:**
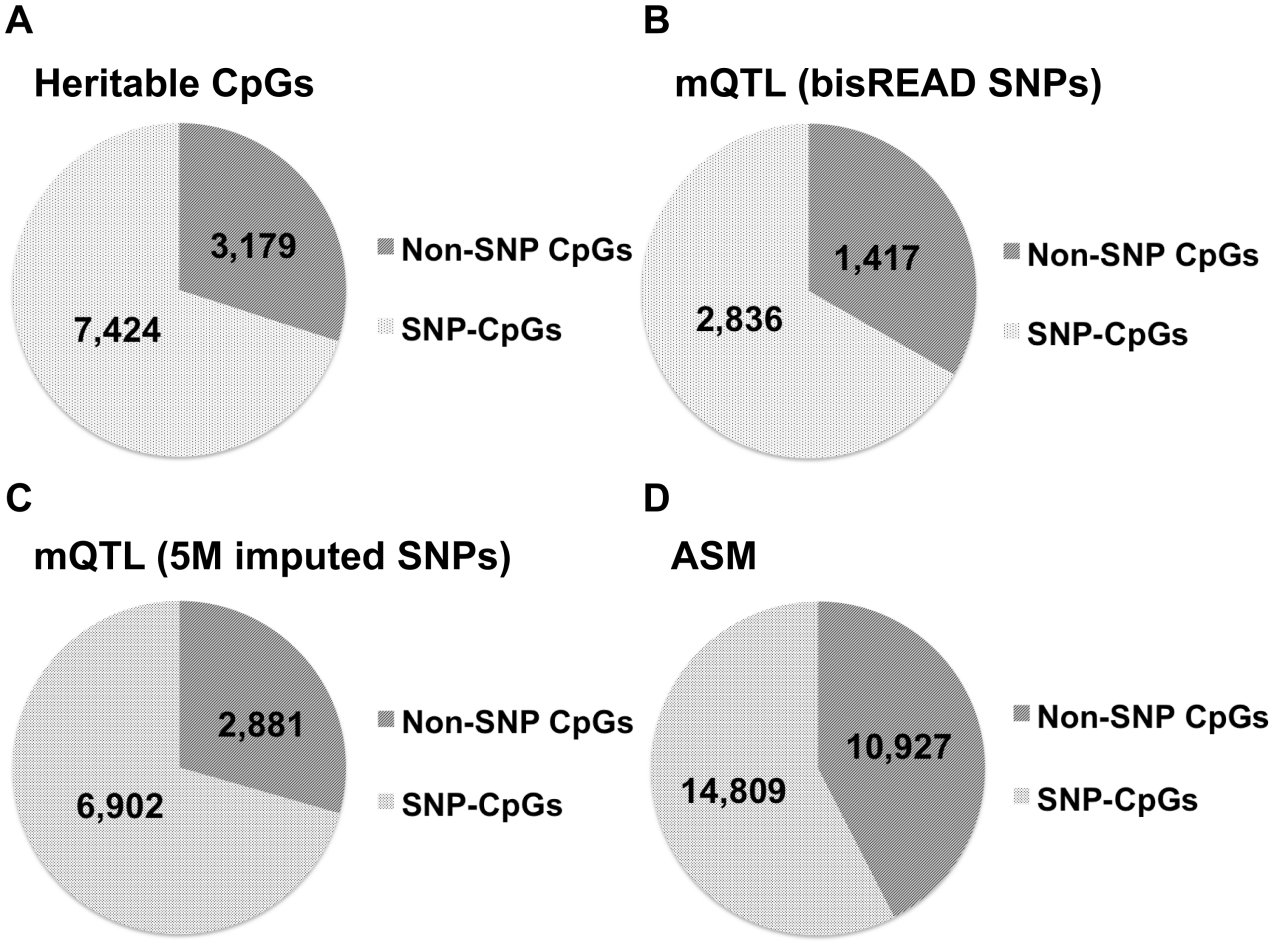
Fraction of non-SNP CpGs and SNP-CpG identified in MPO, mQTL, and ASM analysis. (a) Pie chart showing the number of heritable non-SNP CpGs and heritable SNP-CpGs. (b, c) Pie charts showing the fraction of mQTL associated non-SNP CpG and SNP-CpGs from mQTL analysis using bisREAD SNP data and 5 M imputed SNP array data, respectively. (d) Pie chart showing the fraction of non-SNP CpG ASM and SNP-CpG ASM exist in at least one subject.

We identified a total of 10,593 CpG sites that possessed variable methylation directly correlated with genetic pedigree (Table S1), accounting for ∼13.9% of all variable CpG sites. This result suggests, based on the samples in this study, that genetic factors account for over ten percent of inter-sample DNA methylation variability in human blood. Further analysis revealed that 70% (7,424) of these CpG sites in fact showed variable methylation due to their containing a family-specific SNP at exactly the same locus. This result indicates that the majority of heritable CpG methylation patterns are due to genetic polymorphisms directly altering the substrates of DNA methyltransferases (“SNP-CpGs”), whereas other *cis*- or *trans*- regulatory effects account for only a small fraction (3,169, ∼30%) of heritable CpG methylation (“non-SNP CpGs”) (Figure 2a). Non-SNP CpG sites that localized close by appeared to share similar methylation patterns within individuals of the same family, suggesting that one genetic variant or haplotype could be affecting multiple CpG sites (Table S2, Figure 1b-c). Heritable CpG sites were not enriched for any particular genomic region, as they showed a similar distribution across the genome as all variable CpG sites (Table S3). However, moderate enrichment in gene body and intergenic regions was observed over all characterized CpGs. (Table S3)

### mQTL finds associations between SNPs and CpG sites in a population without trio information

While it is possible to identify heritability in DNA methylation through MPO analysis, for a majority of cases, parent-child trio data is unavailable. In order to determine what fraction of genome-methylome interactions could be identified at a population level when pedigree information was not present, we treated each CpG site as a methylation quantitative trait locus (mQTL), and analyzed the effects on methylation levels of common SNPs or other genetic variants in linkage disequilibrium (LD) with the index SNPs. We sought to perform an analysis using SNP genotypes determined by multiple platforms in order to identify the optimal strategy for identifying genomic contributions to methylation. In some cases, performing additional experiments to obtain sample genotypes is cost-prohibitive; we therefore first utilized the bisulfite sequencing data itself to call genomic SNPs using a previously described method [16]. We obtained genotypes at 15,450 SNP sites after requiring genotypes to be called at putative SNP sites in at least 75% of subjects. Because these SNPs were called only in the captured regions, SNP density was low compared to the whole genome. In order to also perform a more comprehensive mQTL mapping using additional SNPs, we derived SNPs of 57 subjects, a subset of the 96 samples passing quality control of SNP genotyping, using both Affymetrix and Illumina SNP arrays. To avoid platform-specific technical differences, we performed imputation using SNP data from the 1,000 Genomes Project [21], and obtained genotypes for ∼5 million SNPs per sample.

We performed mQTL regression analysis using PLINK with QFAM familial dependence correction [22] between the DNA methylation level of each variable CpG site and the genotypes of SNPs located up to 1 Mb upstream and downstream. Using SNP calls from the bisulfite sequencing data, we identified 7,593 CpG-SNP *cis*-associations at <5% FDR (Table S4), consisting of 4,253 CpG sites associated with 3,842 SNPs. With the ∼5 million genome-wide SNPs, we identified a total of 644,773 CpG-SNP *cis*-associations at <5% FDR (Table S5), consisting of 9,783 CpGs associated with 412,382 SNPs. As in the MPO analysis, a majority of CpG-SNP interactions were due to genetic mutations directly at the CpG site (66.7% and 70.5%, respectively, Figure 2b, 2c).

Generally, the majority of *cis*-regulatory SNPs were located very close to their associated CpG sites in both SNP data sets. For the SNPs called from bisulfite sequencing reads, 47.6% of the CpG-SNP associations were within 2 kb (Table S6, Figure S2a in File S1), and only 15.2% of associations were further away than 100 kb (Table S6, Figure S2b, S2e in File S1). For the SNPs called using genome-wide arrays that more uniformly capture the LD blocks in the human genome, over 64.9% of CpG-SNP associations were within 100 kb (Table S7, Figure S2f in File S1), with the strongest associations mostly within 2 kb (Table S7, Figure S2c in File S1). The identified additional enrichment of short-range CpG-SNP associations in the bisulfite sequencing SNP data appeared to be partially due to sampling bias, because SNPs were called only in captured regions and thus tended to locate very close to CpG sites (Figure S2a, S2e in File S1); it appears that to fully characterize long-range CpG-SNP interactions, SNP genotyping is required. However, bisREAD SNPs can be called directly from methylation sequencing data, whereas SNP genotyping experiments involve extra experimental cost. Additionally, even though the number of bisREAD SNPs used in our analysis was ∼340 fold less than the genome-wide SNPs, it was still possible to identify half of the long-distance non-SNP CpG interactions. Therefore, in cases where SNP genotyping experiments are difficult to perform due to either limited biological material or budgetary constraints, SNPs called from bisulfite sequencing data can still be used to capture a reasonable fraction of *cis*-regulatory interactions, with the caveat that long distance interactions will be under-represented.

Finally, in order to ensure that CpG-SNP interactions were not being missed due to excessive penalties from multiple testing correction in the 5 million SNP case, we additionally performed mQTL analysis using a subset containing 618,580 SNPs in unique LD blocks. The number of CpG-SNP associations decreased to 67,781 (at FDR <5%), indicating that multiple testing penalties were not having a large impact on statistical testing in this case (as a similar fraction of CpG-SNP interactions out of total putative interactions were identified as true in each case).

### ASM finds associations between SNPs and CpGs in single samples

We next used a third strategy to examine the attempt to discern the influence of genetic variation on DNA methylation levels by analyzing allele-specific methylation (ASM). Unlike the MPO and mQTL analysis methods, which utilize information from multiple samples together, ASM examines genome-methylome interactions in one sample at a time. Using this recently developed computational procedure [13], we identified an average of 2,266 variable CpG sites per individual that exhibited significant difference in allelic methylation based on genomic factors (methylation difference >0.2). Consistent with previous observations [12], [13], [23], most ASM events were due to SNPs present directly at CpG sites, (69.7%–92.5%, average 86.4%), with non-SNP CpG sites representing a very small fraction of putative genome-methylome interaction (Figure S3a, S3b in File S1). Additionally, the majority of detected ASM events were present in only a small fraction of subjects (Table S8). After combining all overlapping ASM events, we identified 10,927 and 14,809 ASM events at non-SNP CpGs and SNP-CpGs respectively (Figure 2d). We observed a modest enrichment of ASM on non-SNP CpGs in gene body and intergenic regions (Table S9, Figure S3c, S3d in File S1).

### The efficacy of mQTL and ASM in identifying genome-methylome interaction

While the genomic *cis*-regulated CpG sites identified by MPO appear to be truly heritable through the use of trio information, it remained unclear to what extent mQTL and ASM analyses were characterizing true genome-methylome interactions. We thus next compared the three analyses to determine the efficacy of mQTL and ASM analysis.

**Figure 3:**
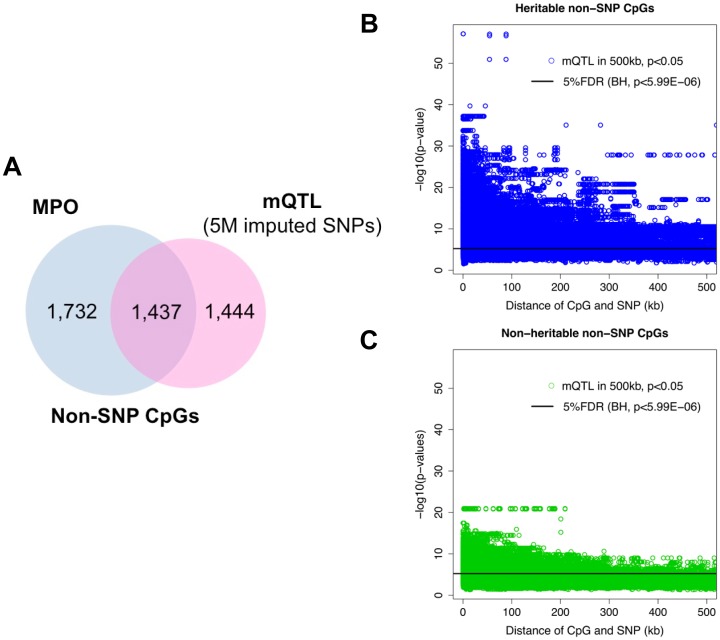
Mapping of CpG sites identified in MPO and mQTL analyses. (a) Venn diagrams showing overlap between non-SNP CpG sites significant in mQTL on 5,257,772 imputed SNPs and heritable CpGs. (b, c) Distribution of heritable CpGs and non-heritable CpGs and associated SNP pair distance within 500kb and their corresponding p-values from mQTL analysis on imputed SNPs.

While, as expected, most SNP-CpG sites identified by mQTL were true positive sites showing heritable CpG methylation (85.3%, Figure S4a in File S1), surprisingly, only 49.9% of non-SNP CpGs identified by mQTL analysis were found heritable by MPO analysis (Figure 3a), indicating that only half of non-SNP CpG sites identified by mQTL mapping are truly heritable. mQTL also failed to identify 54.6% of true heritable non-SNP CpGs (Figure 3a), indicating that for non-SNP CpGs, in addition to having a high false positive rate, mQTL analysis also appears to have a high false negative rate as well. This discrepancy could be due to a number of reasons, including lack of statistical power due to limited sample size, presence of long-range *cis*-interactions at a distance of over 1 megabase and/or *trans*-interactions [24], and the effects of other common or rare alleles not in LD with the SNPs tested. In addition, some marginally significant sites might be included or excluded due to the specific choices of p-value cut-offs for each of the two methods. In fact, when we plotted the mQTL association signals for heritable and non-heritable CpG sites separately, the majority of CpGs most strongly associated with SNPs (low p-value) were heritable CpGs (Figure 3b, Figure S4b in File S1). Non-heritable CpGs in general showed weaker association signals, especially for longer-range *cis*-interactions (Figure 3c, Figure S4c in File S1). It is possible that heritable CpG sites not identified by mQTL analysis could be regulated by other genetic mechanisms.

In contrast to the mQTL analysis, only very small fractions of CpG sites that seemed to exhibit ASM in at least one sample were found to be heritable (5.6% for non-SNP CpGs, 32.6% for SNP-CpGs) (Table S8). One possibility is that calls made by ASM contain a high number of false positive CpG-SNP interactions. However, when we restricted our analysis to the CpG sites that exhibited consistent ASM patterns in two or more individuals, the fractions of sites overlapping with heritable CpGs increased only moderately, and remained far from the 49.9% or 85.3% overlap observed between mQTL calls and heritable CpGs. These calls could be explained by a number of possibilities, including non-genetic parent-of-origin effects (including but not limited to imprinting), random allelic drift [25], environmental factors, potentially higher false positive rates, or higher sensitivity than MPO in detecting allelic differences. Overall, however, ASM appears to have very low specificity in identifying CpG sites regulated by genetic variants.

### Genetic polymorphisms affect the degree of variability in DNA methylation

**Figure 4:**
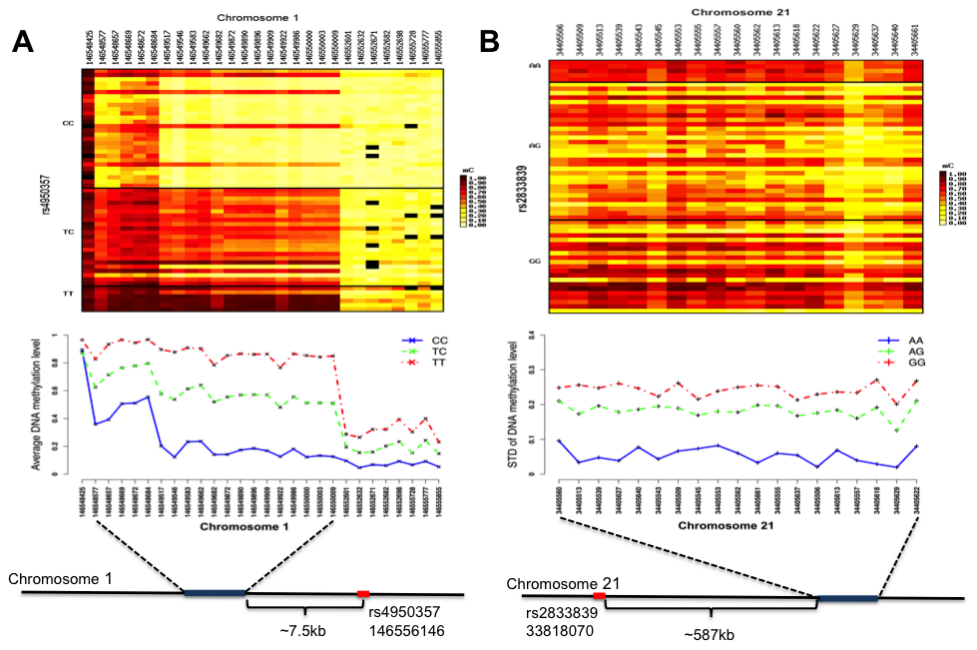
Genotype effects on the mean and variance of DNA methylation (a) Heatmap and line plot showing the association between rs4950357 SNP and the mean methylation of heritable CpGs cluster on chromosome 1 (chr1: 146548425-146555855). (b) The association of rs2833839 vSNP and the variance of methylation on VMR (chr21∶34405506-34405661).

Recently, it was proposed that genetic variants might be regulating the level of variability in molecular phenotypes such as CpG methylation rather than just regulating the exact methylation state [26], [27]. Under this hypothesis, a particular allele of a SNP is associated with highly variable methylation patterns across multiple individuals (Figure 4b) as opposed to being associated with a consistent increase or decrease in mean methylation level (Figure 4a). To determine if variation-SNPs (vSNPs) were present in this data set, we performed a regression analysis on the variance of DNA methylation at each CpG site and the genotypes of nearby SNPs (within 1 Mb). A major technical challenge is that there are only three genotypes for each SNP, and hence the sample size for each regression is limited to three; this could potentially result in a very high false positive rate. To counteract this, we required that a candidate vSNP had a consistent effect on at least five adjacent CpG sites. The false positive rate was estimated to be ∼10% by applying the same procedure to randomly permuted methylation data.

A total of 1,058 genomically-linked variably methylated regions (VMRs) were identified, with many SNPs associated with the variance of multiple nearby CpG sites (Table S10, Figure 4a, 4b). These nearby sites were further grouped into 383 VMR clusters (Table S11) by combining multiple VMRs that were within 100 kb. The majority of VMR clusters (316 clusters, 82.5%) were located within 1 Mb of a set of 438 genes. The largest VMR cluster involved 53 variable CpG sites in a 38 kb region covering GNAS, which is a well documented imprinted gene that has a highly complex expression pattern from both strands [28], [29]. Two other large VMR clusters overlapped with the HoxA gene cluster and protocadherin gamma gene cluster, both of which contain multiple functionally related and co-regulated genes and pseudogenes.

While the full functional consequences of such variable methylation remain largely unknown, we note that very recently four SNPs were found to be associated with rheumatoid arthritis and variance of methylation [18]. In order to test whether the observed VMR clusters could translate into genotype-specific variation at the gene expression level, we examined the top 10 VMR clusters and their respective genes in an array-based whole blood gene expression data set of 240 independent subjects [30]. Nine of the genes within the top ten VMR clusters were expressed at detectable levels (Table 1). Even though the effect sizes were small, we observed three genes (*GNAS*, *PEG3*, and *PCDHGA5*) from different VMR clusters all showing genotype-specific differences contributing to variance at the gene expression level.

**Table 1:** The top 10 VMR clusters and their associated genes.

Number of variable CpGs in VMR clusters	VMR cluster coordinates	Associated genes
53	chr20∶57426730–57464571	**GNAS**, GNAS-AS1
49	chr8∶144358566–144371985	GLI4, **ZNF696**
47	chr7∶27143370–27184750	HOXA2, HOXA3, **HOXA5**, HOXA6, HOXA-AS3
44	chr5∶140718989–140863492	PCDHGA1,PCDHGA2,PCDHGA3,PCDHGA4,**PCDHGA5**,PCDHGA6,PCDHGA7,PCDHGA8,PCDHGA11,PCDHGB1,PCDHGB2,PCDHGB3,**PCDHGB4**,PCDHGB7,PCDHGB8P,PCDHGC3,PCDHGC4
41	chr20∶32255315–32255936	ACTL10,**NECAB3**
35	chr5∶135415001–135416725	VTRNA2-1
28	chr19∶57349099–57352134	MIMT1, **PEG3**, ZIM2
26	chr8∶145162974–145164623	**KIAA1875**, **MAF1**
26	chr11∶7110142–7110456	RBMXL2
24	chr1∶205818899–205819600	PM20D1

The genes in bold text expressed at detectible level in whole blood and were selected for association testing.

## Discussion

In the recent years, association mapping of molecular phenotypes such as gene expression, DNA methylation, or chromatin accessibility as quantitative traits (eQTL, mQTL, dsQTL) has revealed how genetic variants contribute to inter-individual variability and provided additional insights into the modulation of disease susceptibility [1], [20], [31], [32], [33], [34]. The recent technical advances in low-cost genome-wide DNA methylation assays (such as the Illumina 450 k methylation array [35], RRBS [36], and BSPP [16]) have catalyzed a new wave of epigenome-wide association studies aiming to characterize the contribution of both genetic and environmental factors to disease susceptibility [4], [37], with encouraging progress already in sight [18], [38], [39], [40]. However, while new analysis techniques have connected genetic variants, CpG methylation, and disease phenotypes, it remains unclear to what extent we should expect interaction to occur between genetic variation and the variability of DNA methylation, what fraction of interactions are able to be captured with current approaches, and what strategy we should use to efficiently capture these interactions.

In this study, we revealed that a large extent of genome-methylome interaction is completely missed by current analysis methods. By comparing the results from mQTL analysis to MPO analysis, which is guaranteed to find heritable methylation patterns, in 22 nuclear pedigrees, we demonstrated that a large fraction of heritable traits affecting CpG methylation remain hard or impossible to detect with the most widely used analysis method. However, we hypothesize that *trans*-regulation might account for the majority of heritable CpG sites not detectible by conventional mQTL analysis. While the anti-correlation of promoter DNA methylation and gene expression has been observed for many years, the exact mechanistic explanation behind DNA methylation regulating gene expression has yet to be firmly established. More recent observations of positive correlation between gene-body methylation and gene expression have added additional confusion to the functional role of DNA methylation [41], [42], [43], [44]. Stadler et al. recently demonstrated that binding of protein factors to DNA can lead to local reduction of DNA methylation [45], providing the first direct evidence that DNA methylation in general is a passive mark for protein-DNA binding. A corollary of this observation is that a DNA binding protein (such as a transcription factor) for which the expression is an eQTL (i.e. regulated by a genetic variant) can affect DNA methylation levels in hundreds to thousands of its binding regions genome-wide. As such, a single functional variant might regulate many mQTLs, mostly in trans, mediated by its primary effect on a single transcription factor. Connecting these mQTLs to functional variants therefore cannot be accomplished by simple association tests using nearby CpGs and SNPs. Additional information on the transcriptional factors and their direct regulating genes would be required, such as that becoming increasingly available through large-scale ChIP-Seq and DHS mapping efforts like the ENCODE project [46]. A coherent statistical framework for association testing that incorporates the information of protein-DNA binding from genome-wide assays would also be necessary to fully explore genome-methylome interactions.

We also provided a practical assessment on the sensitivity of mQTL mapping at various SNP densities, showing that using over a large number of SNPs can improve the level of statistical significance with diminishing gains in detecting additional SNP-associated CpG sites. On the other hand, for projects based on bisulfite sequencing, the SNP genotypes called from the sequencing reads alone can be used to recover a reasonable fraction of associated CpG sites. As bisulfite sequencing is being widely adopted and algorithms for SNP calling from bisulfite data are being optimized [47], using the smaller number of obtained SNPs could represent an economical option for large-scale EWAS studies, with the understanding that a denser SNP map would still be necessary to recover the majority of long-range regulatory effects.

We additionally characterized the ability of ASM to identify heritable methylation patterns. While we found many CpG sites that both exhibited allele-specific methylation in different individuals and showed heritable methylation patterns across all the pedigrees, the majority of CpG sites identified in our ASM analysis could not be explained by consistent effects of *cis*-regulatory variants across multiple individuals. We reason that ASM analysis is more susceptible to many non-genetic factors, including parent-of-origin effects, random allelic drift, and technical artifacts, and hence might not be appropriate as a primary approach for identifying methylation traits regulated by genetic variants. Population level analysis such as mQTL or MPO (if trio information is available) appears to be necessary to accurately characterize genomic effects on methylation patterns.

Finally, we provide evidence supporting a recently proposed hypothesis that genetic variants can regulate not only the mean but also the variation of molecular phenotypes such as CpG methylation or gene expression. This is not unexpected, as gene regulatory networks are connected through both positive and negative feedback [48], [49]. Reduction of negative feedback has been shown to increase the variability in both prokaryotic and eukaryotic organisms [50], [51], lending mechanistic support to the idea that genetic variants affecting the strength of negative regulation could result in a difference in variability for the components involved in a molecular network. Feinberg and colleagues have proposed that epigenetic variability provides a mechanism for selectable phenotypic variation [27], and provided examples of variable DNA methylation and its role in cancer [26] and rheumatoid arthritis [18]. Although the full extent of variable DNA methylation, as well as its phenotypic consequences, remain to be further characterized with larger cohorts of genetically unrelated individuals, the observation of hundreds of VMRs in the 22 nuclear pedigrees analyzed here suggests that the inherent variability of CpG methylation, and possibly other molecular phenotypes, is likely to play a broad role in human biology and disease.

## Materials and Methods

### Sample collection

Genomic DNAs from the 96 individuals of 22 pedigrees were extracted from whole blood previously collected as part of an on-going genetic study of schizophrenia under the IRB approvals by Utrecht and UCLA. Written consents were obtained from all donors. All personal identifiers were removed and replaced by alpha numerical codes for sample tracking. The information that is available to us as researchers include age, gender and family relationships.

### Targeted bisulfite sequencing with padlock probes

Bisulfite padlock probe design, production and sequencing were previously described [16], [43]. Briefly, genomic DNA was extracted from peripheral blood of 22 pedigrees, and approximately 1 µg of genomic DNA was bisulfite converted with EZ-96 Zymo DNA Methylation-Gold kit (Zymo Research). Approximately 250 ng of bisulfite converted genomic DNAs were mixed with normalized amount of genome-wide scale padlock probes and oligo suppressors. The padlock probes were annealed to bisulfite converted genomic DNA. The gap between two ends of padlock probes was filled and ligated with AmpliTaq DNA polymerase, Stoffel fragment (Life Technologies) and Ampligase (Epicentre), respectively resulting in circularized DNA. The bisulfite sequencing libraries were generated by library-free BSPP protocol as described [16]. Briefly, two-thirds of the circularized DNA of each captured reaction were directly amplified and barcoded with adapter primers compatible with Illumina sequencer. The bisulfite sequencing libraries were purified with AMPure XP magnetic beads (Agencourt), pooled in equimolar ratios, size selected at the size approximately 375 bp with 6% TBE polyacrylamide gel (Life Technologies), and sequenced by Illumina HiSeq2000 and GAIIx sequencers.

### DNA methylation data

The pooled libraries were firstly sequenced with Illumina HiSeq2000 sequencer (100 bp, paired-end reads). Additional sequencings were performed for those samples with number of reads less than 22 millions (53 samples) on the same sequencing libraries with Illumina HiSeq2000 and GAIIx sequencers. Bisulfite sequencing data were processed as described [13], [16]. Briefly, adapter sequences (27 bp from 5′ end) were trimmed from bisulfite reads prior to mapping. In bisulfite sequencing reads, all cytosines were replaced by thymines and mapped to the *in silico* bisulfite converted human genome sequences (hg19) with all cytosines converted to thymines on both strands by bisReadMapper [16]. Absolute DNA methylation level at each CpG site with minimum 10× depth coverage in each sample was calculated at level from 0–1. Summary statistics for sequencing read mapping for all samples sample were reported in Table S12. The quality of the data was assessed by comparing DNA methylation levels at the same CpG sites captured and measured independently on the two strands, which can be treated as internal technical replicates.

### Mid-parent offspring analysis

Mid-parent offspring (MPO) analysis was performed by mid-parent offspring regression [20] to estimate the heritability of DNA methylation at each CpG site. DNA methylation level of the offspring in each trio was compared against the mean DNA methylation level of the parents. In total, 76,408 autosomal variable CpGs (minimum standard deviation of 0.1) shared in at least 80% of subjects were analyzed. The slope of the fitted line was used to estimate the heritability (h^2^) of each CpG site. CpG sites with h^2^ greater than 0.2 in a minimum sample size (number of trio) of 10 were defined as heritable CpGs. The Benjamini-Hochberg method was used to correct for multiple testing errors.

### Methylation quantitative trait loci

Methylation quantitative trait loci (mQTL) analysis was performed by PLINK [22] to determine the association between DNA methylation level of variable CpG sites as described above and SNP genotypes called from methylation data (15,450 SNPs) of 96 subjects or imputed autosomal SNP genotypes (5,257,772 SNPs) of 57 subjects generated by Illumina SNP array (550K) and Affymetrix SNP array. SNP genotypes with a minor allele frequency (MAF) of at least 0.05 and with a Hardy-Weinberg Equilibrium (HWE) p-value >0.001 were included in this analysis. Mendel error rates in each nuclear family with the full trio were calculated by PLINK (Table S13) We used least square linear regression, and the corresponding p-values were calculated for each CpG-SNP association pair within 1 Mb. FDR was calculated by Benjamini-Hochberg multiple correction method to assess the significance of the CpG-SNP association. To deal with family structure, QFAM analysis was performed. 10,000 permutations were performed and p-value was empirically calculated as the fraction of permuted data test-statistic is larger than the non-permuted data test statistic. Additional analyses were performed on subsets of imputed SNPs including 618,580 index SNPs present on Illumina 1 M SNP array. The SNPs that showed strong correlation with DNA methylation were extracted and annotated significant QTL as *cis* if the SNP lay within 1 Mbs of the CpG site.

### SNP imputation

Array genotype data of 96 subjects of this study were generated on two different array platforms, 23 individuals on Illumina SNP array (550K) and 73 individuals on Affymetrix SNP array by Wellcome Trust Case Control Consortium 2 (WTCCC2). After removing poor quality genotyping, there were SNP data of 57 subjects in this study (11 individuals on Illumina SNP array and 46 individual on Affymetrix SNP array). There were 150K of SNP overlapping between the two platforms, so imputation was performed on the two data sets independently. For Illumina SNP data, SNP genotype data from unrelated individuals were phased with Beagle [52] then imputed with Minimac [53] with the 1000 Genomes Project reference [21]. After post-imputation quality control, there were total imputed 8,064,119 SNPs (MAF of 0.01, r^2^ of 0.3). For Affymetrix data set, the SNP genotypes of 43 individuals were imputed with SNP data genotyped on Affymetrix SNP array, including 268 pairs, 236 trios, and 926 unrelated individuals. All Mendel inconsistencies were set to missing before phased with Beagle to take into account family structure. Then Minimac was used for imputation. There were 8,022,142 SNPs after the post-imputation quality control. Approximately 7,800,000 overlapping SNPs between the two imputed data sets were merged by including only well imputed SNPs on the two data sets. SNPs with MAF >0.05 and HWE >0.001 were extracted, and there were 5,257,772 imputed SNPs remained in this study.

### Allele-specific methylation

Allele-specific methylation (ASM) analysis was performed as described [13]. Briefly, we generated the 2×2 contingency table where the two columns containing the two alleles and the two rows containing the counts of methylated and un-methylated cytosines at CpG site(s) on the read containing heterozygous SNP(s). The p-value at each CpG site was calculated by Fisher's exact test. We identified ASM if the p-value was less than 0.001 and the methylation frequency between the two alleles was greater than 0.2.

### Genomic region annotation

Genomic features of CpG sites were assigned using bedtools [54] according to genomic annotation structure described by Bikikova et al, 2011 [35]. The enrichment of CpG sites from different analyses was calculated as the ratio between significant CpG sites from each analysis and CpG sites included in the analysis.

### Variation-SNP and variably mathylated regions

We identified vSNPs and VMRs by performing association tests. Linear regression was performed on the variance of DNA methylation at each CpG site among individuals and the three genotype groups (AA, AB, BB) within 1 Mb distance. The t-score of each CpG-SNP pair was calculated, and the false discovery rate was calculated by using different cutoff values for the test statistic values. To deal with the high rate of false positive signals, we required at least five adjacent CpG sites with maximal spacing 200 bp between CpGs showing consistent association for VMRs. We then grouped the overlapping or adjacent VMRs into clusters. We note that VMRs associated with different vSNPs could be partially overlapping, so they could be grouped into the same cluster.

### Accession number

DNA methylation data of this study has been deposited in the Gene Expression Omnibus (GEO) database under accession number GSE47614.

## Supporting Information

Table S1 All heritable CpG list.
(XLSX)

Table S2 Heritable non-SNP CpG clusters.
(XLSX)

Table S3 Distribution of heritable CpG sites based on genomic regions (percentage).
(DOCX)

Table S4 mQTL hits (bisREAD SNPs).
(XLSX)

Table S5 mQTL hits (5 M imputed SNPs).
(XLSX)

Table S6 Distribution of CpG and SNP associations at different distance between CpG and SNP pairs (bisREAD SNPs).
(DOCX)

Table S7 Distribution of CpG and SNP associations at different distance between CpG and SNP pairs (5 M imputed SNPs).
(DOCX)

Table S8 Number of non-SNP CpGs showing ASM shared by multiple individuals and the overlap with heritable CpGs.
(DOCX)

Table S9 Genomic region annotation of CpG ASM (percentage).
(DOCX)

Table S10 VMRs and their associated vSNPs.
(XLSX)

Table S11 VMR clusters.
(XLSX)

Table S12 Bisulfite sequencing and mapping summary.
(XLSX)

Table S13 Mendel error rates of SNP genotypes.
(DOCX)

File S1 **Figure S1. Hierarchical clustering of high variable CpGs. Figure S2. Manhattan and density plots showing the distribution of associated CpG and SNP pairs across all chromosomes between CpG and SNP pair of 0-2kb (left) and 100kb-1Mb (right) of mQTL analysis using bisREAD SNP data (a, b) and 5M imputed SNP data (c, d), respectively.** Distribution of CpG and SNP associations and their corresponding absolute distances of mQTL analysis using bisREAD SNP data (e) and 5M imputed SNP data (f), respectively. **Figure S3. Examples of ASM events and regional annotation of CpG associated with ASM.** (a, b) Example of allele specific DNA methylation of non-SNP CpG and SNP-CpG, respectively. (b) The presence of T SNP on CpG sites disrupted DNA methylation of that allele. (c, d) Pie charts showing the distribution of non-SNP CpG ASM and SNP-CpG ASM, respectively, in different regions. **Figure S4.** (a) Venn diagrams showing overlap between SNP-CpG significant in mQTL and MPO analyses (based on the 5M imputed SNPs). (b, c) Distribution of heritable CpG and non- heritable CpGs, respectively, and SNP pair in mQTL analysis within 500kb and their corresponding p-values.
(PDF)

## References

[1] McDaniell R, Lee BK, Song L, Liu Z, Boyle AP, et al (2010) Heritable individual-specific and allele-specific chromatin signatures in humans. Science 328: 235–239. 20299549 10.1126/science.1184655 PMC2929018

[2] Zhang Y, Rohde C, Reinhardt R, Voelcker-Rehage C, Jeltsch A (2009) Non-imprinted allele-specific DNA methylation on human autosomes. Genome biology 10: R138. 19958531 10.1186/gb-2009-10-12-r138 PMC2812945

[3] Schalkwyk LC, Meaburn EL, Smith R, Dempster EL, Jeffries AR, et al (2010) Allelic skewing of DNA methylation is widespread across the genome. American journal of human genetics 86: 196–212. 20159110 10.1016/j.ajhg.2010.01.014 PMC2820163

[4] Rakyan VK, Down TA, Balding DJ, Beck S (2011) Epigenome-wide association studies for common human diseases. Nature reviews Genetics 12: 529–541. 10.1038/nrg3000 PMC3508712 21747404

[5] Hannum G, Guinney J, Zhao L, Zhang L, Hughes G, et al (2013) Genome-wide methylation profiles reveal quantitative views of human aging rates. Molecular cell 49: 359–367. 23177740 10.1016/j.molcel.2012.10.016 PMC3780611

[6] Tycko B (2010) Allele-specific DNA methylation: beyond imprinting. Human molecular genetics 19: R210–220. 20855472 10.1093/hmg/ddq376 PMC2953749

[7] Feinberg AP (2007) Phenotypic plasticity and the epigenetics of human disease. Nature 447: 433–440. 17522677 10.1038/nature05919

[8] Bell JT, Pai AA, Pickrell JK, Gaffney DJ, Pique-Regi R, et al (2011) DNA methylation patterns associate with genetic and gene expression variation in HapMap cell lines. Genome biology 12: R10. 21251332 10.1186/gb-2011-12-1-r10 PMC3091299

[9] Fraser HB, Lam LL, Neumann SM, Kobor MS (2012) Population-specificity of human DNA methylation. Genome biology 13: R8. 22322129 10.1186/gb-2012-13-2-r8 PMC3334571

[10] Gertz J, Varley KE, Reddy TE, Bowling KM, Pauli F, et al (2011) Analysis of DNA methylation in a three-generation family reveals widespread genetic influence on epigenetic regulation. PLoS genetics 7: e1002228. 21852959 10.1371/journal.pgen.1002228 PMC3154961

[11] van Eijk KR, de Jong S, Boks MP, Langeveld T, Colas F, et al (2012) Genetic analysis of DNA methylation and gene expression levels in whole blood of healthy human subjects. BMC genomics 13: 636. 23157493 10.1186/1471-2164-13-636 PMC3583143

[12] Hellman A, Chess A (2010) Extensive sequence-influenced DNA methylation polymorphism in the human genome. Epigenetics & chromatin 3: 11. 20497546 10.1186/1756-8935-3-11 PMC2893533

[13] Shoemaker R, Deng J, Wang W, Zhang K (2010) Allele-specific methylation is prevalent and is contributed by CpG-SNPs in the human genome. Genome research 20: 883–889. 20418490 10.1101/gr.104695.109 PMC2892089

[14] Fang F, Hodges E, Molaro A, Dean M, Hannon GJ, et al (2012) Genomic landscape of human allele-specific DNA methylation. Proceedings of the National Academy of Sciences of the United States of America 109: 7332–7337. 22523239 10.1073/pnas.1201310109 PMC3358917

[15] Schilling E, El Chartouni C, Rehli M (2009) Allele-specific DNA methylation in mouse strains is mainly determined by cis-acting sequences. Genome research 19: 2028–2035. 19687144 10.1101/gr.095562.109 PMC2775599

[16] Diep D, Plongthongkum N, Gore A, Fung HL, Shoemaker R, et al (2012) Library-free methylation sequencing with bisulfite padlock probes. Nature methods 9: 270–272. 22306810 10.1038/nmeth.1871 PMC3461232

[17] Houseman EA, Accomando WP, Koestler DC, Christensen BC, Marsit CJ, et al (2012) DNA methylation arrays as surrogate measures of cell mixture distribution. BMC Bioinformatics 13: 86. 22568884 10.1186/1471-2105-13-86 PMC3532182

[18] Liu Y, Aryee MJ, Padyukov L, Fallin MD, Hesselberg E, et al (2013) Epigenome-wide association data implicate DNA methylation as an intermediary of genetic risk in rheumatoid arthritis. Nature biotechnology 31: 142–147. 10.1038/nbt.2487 PMC3598632 23334450

[19] Jaffe AE, Irizarry RA (2014) Accounting for cellular heterogeneity is critical in epigenome-wide association studies. Genome biology 15: R31. 24495553 10.1186/gb-2014-15-2-r31 PMC4053810

[20] Stranger BE, Nica AC, Forrest MS, Dimas A, Bird CP, et al (2007) Population genomics of human gene expression. Nat Genet 39: 1217–1224. 17873874 10.1038/ng2142 PMC2683249

[21] Abecasis GR, Altshuler D, Auton A, Brooks LD, Durbin RM, et al (2010) A map of human genome variation from population-scale sequencing. Nature 467: 1061–1073. 20981092 10.1038/nature09534 PMC3042601

[22] Purcell S, Neale B, Todd-Brown K, Thomas L, Ferreira MA, et al (2007) PLINK: a tool set for whole-genome association and population-based linkage analyses. American journal of human genetics 81: 559–575. 17701901 10.1086/519795 PMC1950838

[23] Xie W, Barr CL, Kim A, Yue F, Lee AY, et al (2012) Base-resolution analyses of sequence and parent-of-origin dependent DNA methylation in the mouse genome. Cell 148: 816–831. 22341451 10.1016/j.cell.2011.12.035 PMC3343639

[24] Greaves I, Groszmann M, Dennis ES, Peacock WJ (2012) Trans-chromosomal methylation. Epigenetics: official journal of the DNA Methylation Society 7: 800–805. 10.4161/epi.20820 PMC3427274 22705969

[25] Gimelbrant A, Hutchinson JN, Thompson BR, Chess A (2007) Widespread monoallelic expression on human autosomes. Science 318: 1136–1140. 18006746 10.1126/science.1148910

[26] Hansen KD, Timp W, Bravo HC, Sabunciyan S, Langmead B, et al (2011) Increased methylation variation in epigenetic domains across cancer types. Nature genetics 43: 768–775. 21706001 10.1038/ng.865 PMC3145050

[27] Feinberg AP, Irizarry RA (2010) Evolution in health and medicine Sackler colloquium: Stochastic epigenetic variation as a driving force of development, evolutionary adaptation, and disease. Proceedings of the National Academy of Sciences of the United States of America 107 Suppl 1 1757–1764. 20080672 10.1073/pnas.0906183107 PMC2868296

[28] Bastepe M (2007) The GNAS Locus: Quintessential Complex Gene Encoding Gsalpha, XLalphas, and other Imprinted Transcripts. Current genomics 8: 398–414. 19412439 10.2174/138920207783406488 PMC2671723

[29] Plagge A, Kelsey G (2006) Imprinting the Gnas locus. Cytogenetic and genome research 113: 178–187. 16575178 10.1159/000090830

[30] Luykx JJ, Bakker SC, Lentjes E, Neeleman M, Strengman E, et al.. (2013) Genome-wide association study of monoamine metabolite levels in human cerebrospinal fluid. Molecular psychiatry. 10.1038/mp.2012.183 23319000

[31] Degner JF, Pai AA, Pique-Regi R, Veyrieras JB, Gaffney DJ, et al (2012) DNase I sensitivity QTLs are a major determinant of human expression variation. Nature 482: 390–394. 22307276 10.1038/nature10808 PMC3501342

[32] Pickrell JK, Marioni JC, Pai AA, Degner JF, Engelhardt BE, et al (2010) Understanding mechanisms underlying human gene expression variation with RNA sequencing. Nature 464: 768–772. 20220758 10.1038/nature08872 PMC3089435

[33] Zhang D, Cheng L, Badner JA, Chen C, Chen Q, et al (2010) Genetic control of individual differences in gene-specific methylation in human brain. American journal of human genetics 86: 411–419. 20215007 10.1016/j.ajhg.2010.02.005 PMC2833385

[34] Emilsson V, Thorleifsson G, Zhang B, Leonardson AS, Zink F, et al (2008) Genetics of gene expression and its effect on disease. Nature 452: 423–428. 18344981 10.1038/nature06758

[35] Bibikova M, Barnes B, Tsan C, Ho V, Klotzle B, et al (2011) High density DNA methylation array with single CpG site resolution. Genomics 98: 288–295. 21839163 10.1016/j.ygeno.2011.07.007

[36] Boyle P, Clement K, Gu H, Smith ZD, Ziller M, et al (2012) Gel-free multiplexed reduced representation bisulfite sequencing for large-scale DNA methylation profiling. Genome biology 13: R92. 23034176 10.1186/gb-2012-13-10-r92 PMC3491420

[37] Teperino R, Lempradl A, Pospisilik JA (2013) Bridging epigenomics and complex disease: the basics. Cellular and molecular life sciences: CMLS. 10.1007/s00018-013-1299-z PMC11113658 23463237

[38] Drong AW, Nicholson G, Hedman AK, Meduri E, Grundberg E, et al (2013) The presence of methylation quantitative trait Loci indicates a direct genetic influence on the level of DNA methylation in adipose tissue. PLoS One 8: e55923. 23431366 10.1371/journal.pone.0055923 PMC3576415

[39] Shenker NS, Polidoro S, van Veldhoven K, Sacerdote C, Ricceri F, et al (2013) Epigenome-wide association study in the European Prospective Investigation into Cancer and Nutrition (EPIC-Turin) identifies novel genetic loci associated with smoking. Human molecular genetics 22: 843–851. 23175441 10.1093/hmg/dds488

[40] Joubert BR, Haberg SE, Nilsen RM, Wang X, Vollset SE, et al (2012) 450K epigenome-wide scan identifies differential DNA methylation in newborns related to maternal smoking during pregnancy. Environmental health perspectives 120: 1425–1431. 22851337 10.1289/ehp.1205412 PMC3491949

[41] Ball MP, Li JB, Gao Y, Lee JH, LeProust EM, et al (2009) Targeted and genome-scale strategies reveal gene-body methylation signatures in human cells. Nature biotechnology 27: 361–368. 10.1038/nbt.1533 PMC3566772 19329998

[42] Jones PA (2012) Functions of DNA methylation: islands, start sites, gene bodies and beyond. Nature reviews Genetics 13: 484–492. 10.1038/nrg3230 22641018

[43] Deng J, Shoemaker R, Xie B, Gore A, LeProust EM, et al (2009) Targeted bisulfite sequencing reveals changes in DNA methylation associated with nuclear reprogramming. Nat Biotechnol 27: 353–360. 19330000 10.1038/nbt.1530 PMC2715272

[44] Lister R, Pelizzola M, Dowen RH, Hawkins RD, Hon G, et al (2009) Human DNA methylomes at base resolution show widespread epigenomic differences. Nature 462: 315–322. 19829295 10.1038/nature08514 PMC2857523

[45] Stadler MB, Murr R, Burger L, Ivanek R, Lienert F, et al (2011) DNA-binding factors shape the mouse methylome at distal regulatory regions. Nature 480: 490–495. 22170606 10.1038/nature10716

[46] Dunham I, Kundaje A, Aldred SF, Collins PJ, Davis CA, et al (2012) An integrated encyclopedia of DNA elements in the human genome. Nature 489: 57–74. 22955616 10.1038/nature11247 PMC3439153

[47] Liu Y, Siegmund KD, Laird PW, Berman BP (2012) Bis-SNP: Combined DNA methylation and SNP calling for Bisulfite-seq data. Genome biology 13: R61. 22784381 10.1186/gb-2012-13-7-r61 PMC3491382

[48] Hartwell LH, Hopfield JJ, Leibler S, Murray AW (1999) From molecular to modular cell biology. Nature 402: C47–52. 10591225 10.1038/35011540

[49] Milo R, Shen-Orr S, Itzkovitz S, Kashtan N, Chklovskii D, et al (2002) Network motifs: simple building blocks of complex networks. Science 298: 824–827. 12399590 10.1126/science.298.5594.824

[50] Becskei A, Serrano L (2000) Engineering stability in gene networks by autoregulation. Nature 405: 590–593. 10850721 10.1038/35014651

[51] Raj A, Rifkin SA, Andersen E, van Oudenaarden A (2010) Variability in gene expression underlies incomplete penetrance. Nature 463: 913–918. 20164922 10.1038/nature08781 PMC2836165

[52] Browning BL, Browning SR (2009) A unified approach to genotype imputation and haplotype-phase inference for large data sets of trios and unrelated individuals. American journal of human genetics 84: 210–223. 19200528 10.1016/j.ajhg.2009.01.005 PMC2668004

[53] Howie B, Fuchsberger C, Stephens M, Marchini J, Abecasis GR (2012) Fast and accurate genotype imputation in genome-wide association studies through pre-phasing. Nat Genet 44: 955–959. 22820512 10.1038/ng.2354 PMC3696580

[54] Quinlan AR, Hall IM (2010) BEDTools: a flexible suite of utilities for comparing genomic features. Bioinformatics 26: 841–842. 20110278 10.1093/bioinformatics/btq033 PMC2832824

