# Supplementary material for: Characterization of Genome-Methylome Interactions in 22 Nuclear Pedigrees
Source: PLoS One. 2014 Jul 14;9(7):e99313. doi: 10.1371/journal.pone.0099313 (PMC4096397; doi:10.1371/journal.pone.0099313)
Supplement: Table S3 — Distribution of heritable CpG sites based on genomic regions (percentage). (DOCX) [file pone.0099313.s003.docx]

**Table S3.** Distribution of heritable CpG sites based on genomic regions (percentage).

| **Methylation data** | **TSS1500** | **TSS200** | **5' UTR** | **First exon** | **Gene body** | **3' UTR** | **Intergenic** |
| --- | --- | --- | --- | --- | --- | --- | --- |
| heritable non-SNP CpGs | 9.39 | 2.59 | 15.71 | 5.48 | 42.85 | 5.60 | 18.37 |
| heritable SNP-CpGs | 5.91 | 0.92 | 12.64 | 3.47 | 53.77 | 5.72 | 17.58 |
| variable CpGs (min STD 0.1) | 10.98 | 1.74 | 13.85 | 5.77 | 44.72 | 5.59 | 17.34 |
| all characterized CpGs | 12.15 | 4.30 | 15.43 | 8.77 | 40.24 | 5.52 | 13.60 |
