# Supplementary material for: Characterization of Genome-Methylome Interactions in 22 Nuclear Pedigrees
Source: PLoS One. 2014 Jul 14;9(7):e99313. doi: 10.1371/journal.pone.0099313 (PMC4096397; doi:10.1371/journal.pone.0099313)
Supplement: Table S6 — Distribution of CpG and SNP associations at different distance between CpG and SNP pairs (bisREAD SNPs). (DOCX) [file pone.0099313.s006.docx]

**Table S6.** Distribution of CpG and SNP associations at different distance between CpG and SNP pairs (bisREAD SNPs).

| Distance of CpG and SNP | Number of associations | % of total number of associations |
| --- | --- | --- |
| 0-2kb | 1,071 | 47.6 |
| 0-10kb | 1,331 | 59.2 |
| 10-20kb | 142 | 6.3 |
| 20-30kb | 102 | 4.5 |
| 30-40kb | 84 | 3.7 |
| 40-50kb | 51 | 2.3 |
| 0-100kb | 1,907 | 84.8 |
| 0-150kb | 1,986 | 88.3 |
| 100kb-1Mb | 341 | 15.2 |
| 150kb-1Mb | 262 | 11.7 |
