# Supplementary material for: Characterization of Genome-Methylome Interactions in 22 Nuclear Pedigrees
Source: PLoS One. 2014 Jul 14;9(7):e99313. doi: 10.1371/journal.pone.0099313 (PMC4096397; doi:10.1371/journal.pone.0099313)
Supplement: Table S7 — Distribution of CpG and SNP associations at different distance between CpG and SNP pairs (5 M imputed SNPs). (DOCX) [file pone.0099313.s007.docx]

**Table S7.** Distribution of CpG and SNP associations at different distance between CpG and SNP pairs (5M imputed SNPs).

| Distance of CpG and SNP | Number of associations | % of total number of associations |
| --- | --- | --- |
| 0-2kb | 9,325 | 6.4 |
| 0-10kb | 29,052 | 20.1 |
| 10-20kb | 15,648 | 10.8 |
| 20-30kb | 11,512 | 8.0 |
| 30-40kb | 8,669 | 6.0 |
| 40-50kb | 7,003 | 4.8 |
| 0-100kb | 93,960 | 64.9 |
| 0-150kb | 105,711 | 73.0 |
| 100kb-1Mb | 50,820 | 35.1 |
| 150kb-1Mb | 39,069 | 27.0 |
