# Supplementary material for: Characterization of Genome-Methylome Interactions in 22 Nuclear Pedigrees
Source: PLoS One. 2014 Jul 14;9(7):e99313. doi: 10.1371/journal.pone.0099313 (PMC4096397; doi:10.1371/journal.pone.0099313)
Supplement: Table S8 — Number of non-SNP CpGs showing ASM shared by multiple individuals and the overlap with heritable CpGs. (DOCX) [file pone.0099313.s008.docx]

**Table S8.** Number of non-SNP CpGs showing ASM shared by multiple individuals and the overlap with heritable CpGs.

| # of subjects | # of ASM CpGs | # of ASM CpGs found heritable | % of ASM CpGs found heritable | # of ASM CpGs found variable | % of ASM CpGs found heritable & variable |
| --- | --- | --- | --- | --- | --- |
| 1 | 6,079 | 97 | 1.60 | 1745 | 5.56 |
| 2 | 2,005 | 65 | 3.24 | 775 | 8.39 |
| 3 | 918 | 43 | 4.68 | 385 | 11.17 |
| 4 | 458 | 41 | 8.95 | 208 | 19.71 |
| 5 | 297 | 29 | 9.76 | 148 | 19.59 |
| 6 | 216 | 23 | 10.65 | 114 | 20.18 |
| 7 | 148 | 26 | 17.57 | 84 | 30.95 |
| 8 | 104 | 23 | 22.12 | 58 | 39.66 |
| 9 | 91 | 24 | 26.37 | 56 | 42.86 |
| 10 | 70 | 22 | 31.43 | 49 | 44.90 |
| 11 | 56 | 19 | 33.93 | 39 | 48.72 |
| 12 | 39 | 11 | 28.21 | 27 | 40.74 |
| 13 | 37 | 10 | 27.03 | 24 | 41.67 |
| 14 | 43 | 17 | 39.53 | 28 | 60.71 |
| 15 | 35 | 11 | 31.43 | 26 | 42.31 |
