# Supplementary material for: Characterization of Genome-Methylome Interactions in 22 Nuclear Pedigrees
Source: PLoS One. 2014 Jul 14;9(7):e99313. doi: 10.1371/journal.pone.0099313 (PMC4096397; doi:10.1371/journal.pone.0099313)
Supplement: Table S9 — Genomic region annotation of CpG ASM (percentage). (DOCX) [file pone.0099313.s009.docx]

**Table S9**. Genomic region annotation of CpG ASM (percentage)

| **Methylation data** | **TSS1500** | **TSS200** | **5' UTR** | **First exon** | **Gene body** | **3' UTR** | **Intergenic** |
| --- | --- | --- | --- | --- | --- | --- | --- |
| Non-SNP CpG ASM | 10.08 | 1.46 | 14.89 | 4.51 | 41.25 | 5.33 | 22.48 |
| SNP-CpG ASM | 6.19 | 1.09 | 12.45 | 2.75 | 54.01 | 5.56 | 17.95 |
| all captured CpGs | 12.2 | 4.6 | 15.8 | 9.0 | 38.8 | 5.3 | 14.3 |
