# Supplementary material for: Characterization of Genome-Methylome Interactions in 22 Nuclear Pedigrees
Source: PLoS One. 2014 Jul 14;9(7):e99313. doi: 10.1371/journal.pone.0099313 (PMC4096397; doi:10.1371/journal.pone.0099313)
Supplement: Table S13 — Mendel error rates of SNP genotypes. (DOCX) [file pone.0099313.s013.docx]

**Table S13.** Mendel error rates of SNP genotypes.

bisREAD SNPs Mendel error rate

| Family IDs | CHLD | N | Mendel error rate |
| --- | --- | --- | --- |
| FAM1 | 2 | 890 | 0.058 |
| FAM2 | 2 | 1944 | 0.126 |
| FAM3 | 3 | 390 | 0.025 |
| FAM4 | 2 | 616 | 0.040 |
| FAM5 | 3 | 429 | 0.028 |
| FAM6 | 2 | 345 | 0.022 |
| FAM7 | 3 | 701 | 0.045 |
| FAM8 | 3 | 848 | 0.055 |
| FAM9 | 2 | 437 | 0.028 |
| FAM10 | 2 | 204 | 0.013 |
| FAM11 | 2 | 342 | 0.022 |
| FAM12 | 3 | 396 | 0.026 |
| FAM13 | 3 | 562 | 0.036 |
| FAM14 | 3 | 1000 | 0.065 |
| FAM15 | 2 | 431 | 0.028 |
| FAM16 | 2 | 645 | 0.042 |
| FAM17 | 2 | 300 | 0.019 |
| FAM18 | 2 | 835 | 0.054 |
| FAM19 | 3 | 466 | 0.030 |
| FAM20 | 2 | 281 | 0.018 |
| FAM21 | 2 | 233 | 0.015 |
| FAM22 | 2 | 785 | 0.051 |

5M Imputed SNPs Mendel error rate

| Family IDs | CHLD | N | Mendel error rate |
| --- | --- | --- | --- |
| FAM6 | 1 | 293 | 5.573E-05 |
| FAM18 | 1 | 161 | 3.062E-05 |
| FAM11 | 1 | 115 | 2.187E-05 |
| FAM12 | 2 | 800 | 1.522E-04 |
| FAM5 | 1 | 3013 | 5.731E-04 |
| FAM1 | 2 | 7631 | 1.451E-03 |

CHLD: number of offspring in each family, N: number of Mendel error in each family
