# Supplementary material for: Characterization of Genome-Methylome Interactions in 22 Nuclear Pedigrees
Source: PLoS One. 2014 Jul 14;9(7):e99313. doi: 10.1371/journal.pone.0099313 (PMC4096397; doi:10.1371/journal.pone.0099313)

Figure S1. Hierarchical clustering of high variable CpGs.

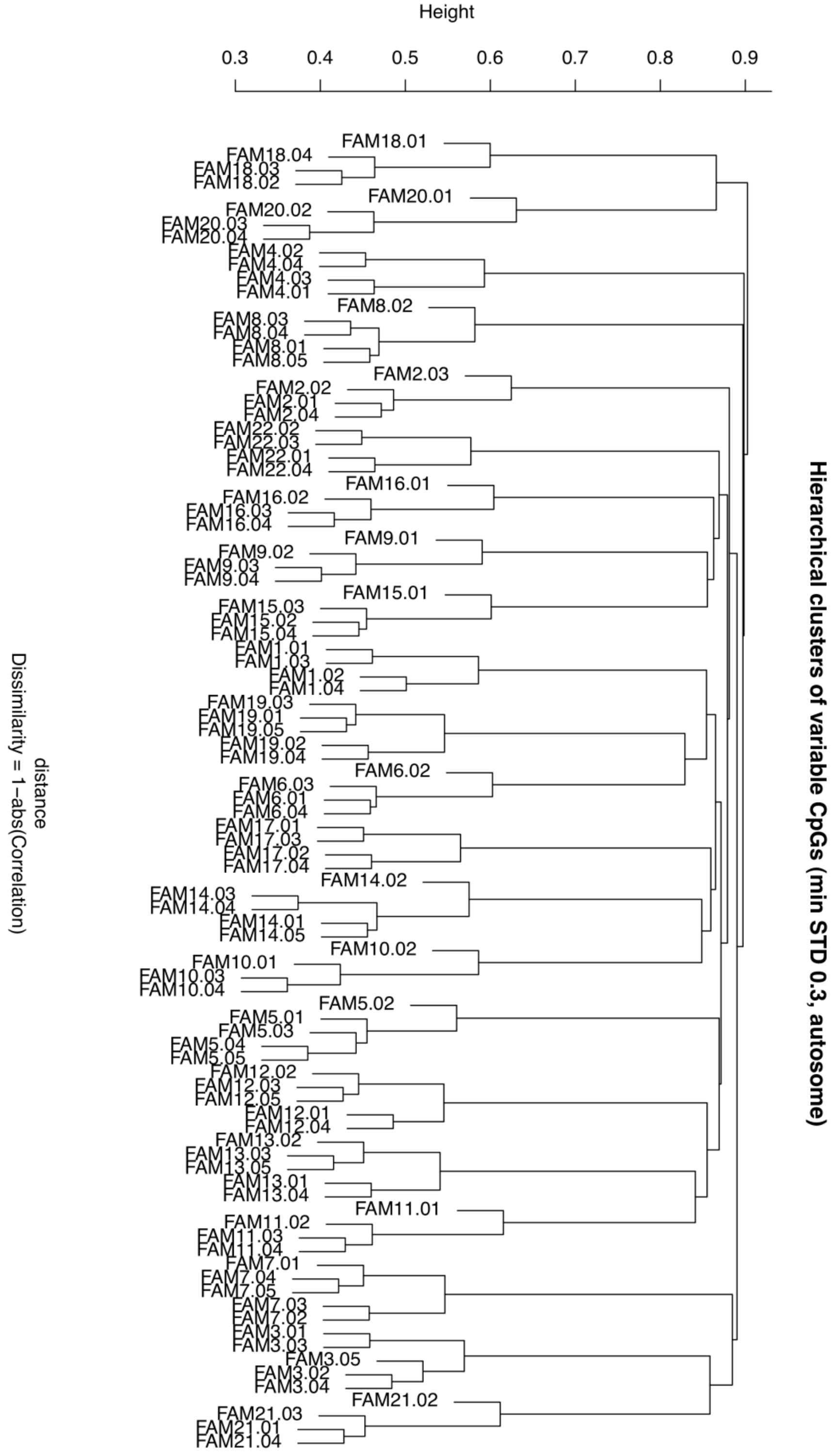

Figure S2. Manhattan and density plots showing the distribution of associated CpG and SNP pairs across all chromosomes between CpG and SNP pair of 0-2kb (left) and 100kb-1Mb (right) of mQTL analysis using bisREAD SNP data (a, b) and 5M imputed SNP data (c, d), respectively. Distribution of CpG and SNP associations and their corresponding absolute distances of mQTL analysis using bisREAD SNP data (e) and 5M imputed SNP data (f), respectively.

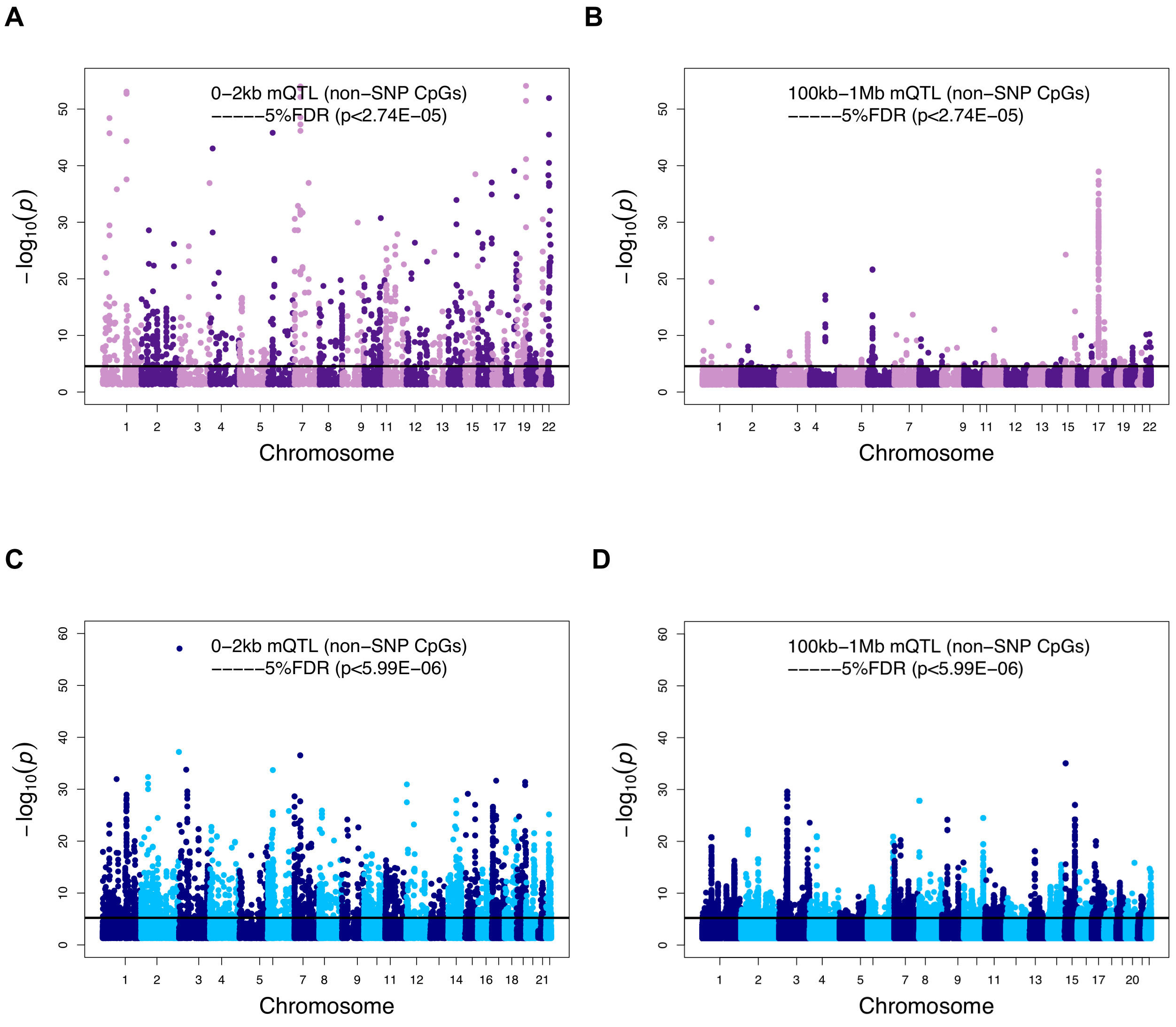

E

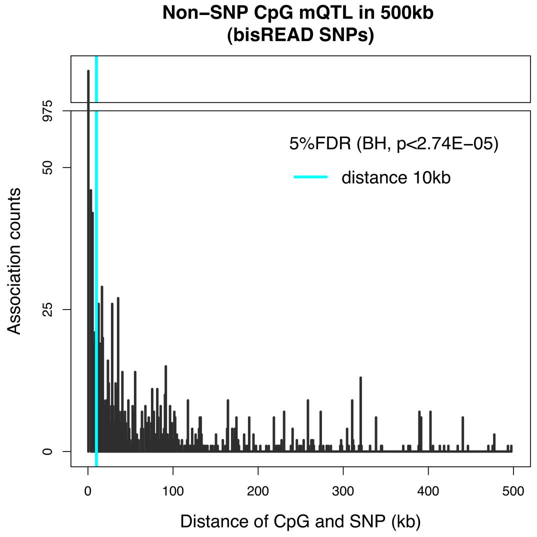
Non−SNP CpG mQTL in 500kb
(bisREAD SNPs)
5%FDR (BH, p<2.74E−05)
distance 10kb
Association counts
0
25
50
975
0
100
200
300
400
500
Distance of CpG and SNP (kb)

F

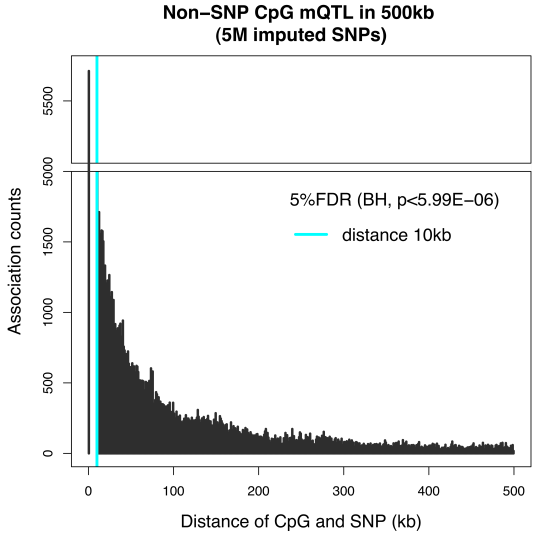
Non−SNP CpG mQTL in 500kb
(5M imputed SNPs)
5%FDR (BH, p<5.99E−06)
distance 10kb
Association counts
0
500
1000
1500
5000
5500
0
100
200
300
400
500
Distance of CpG and SNP (kb)

Figure S3. Examples of ASM events and regional annotation of CpG associated with ASM. (a, b) Example of allele specific DNA methylation of non-SNP CpG and SNP-CpG, respectively. (b) The presence of T SNP on CpG sites disrupted DNA methylation of that allele. (c, d) Pie charts showing the distribution of non-SNP CpG ASM and SNP-CpG ASM, respectively, in different regions.

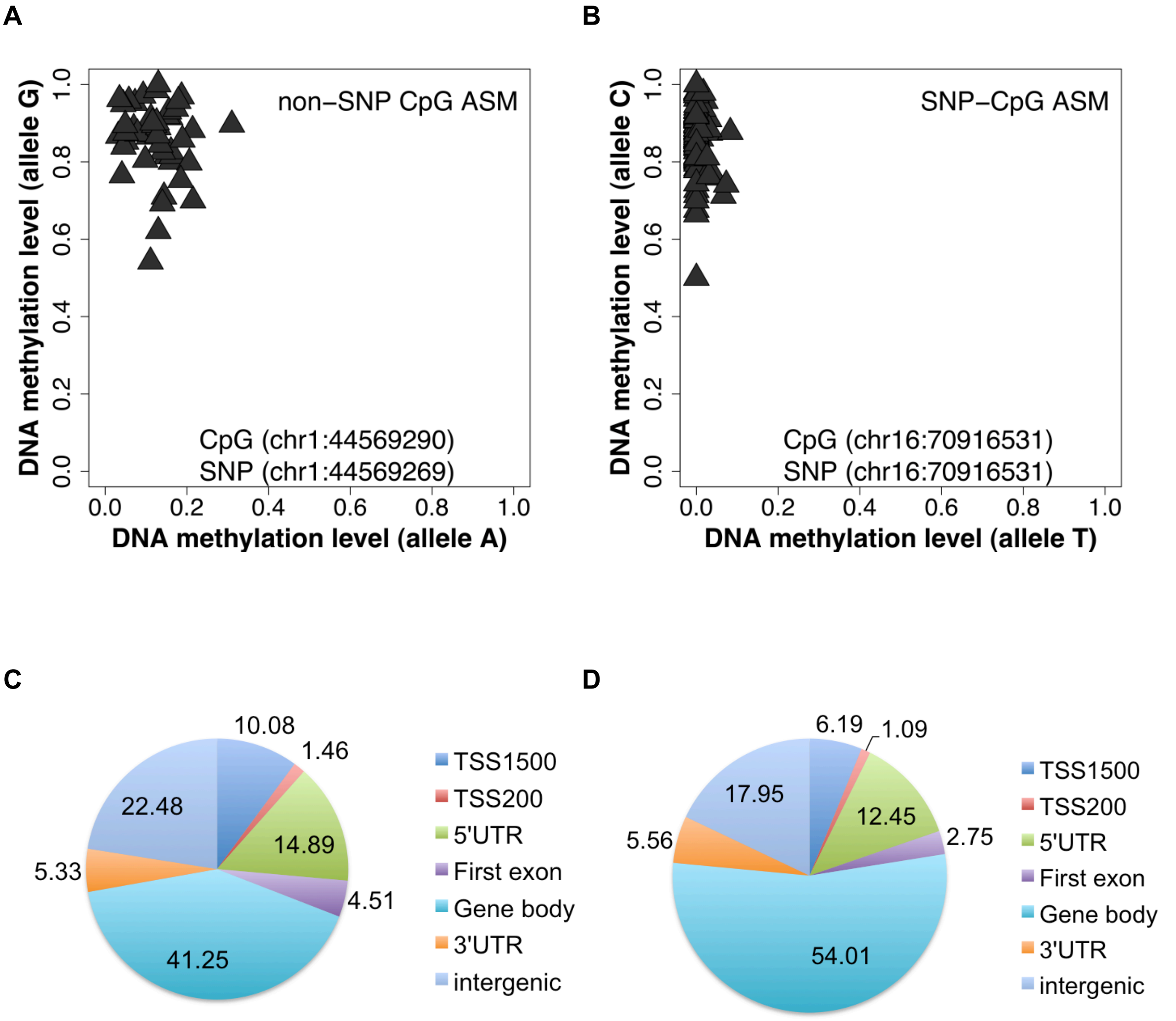

Figure S4. (a) Venn diagrams showing overlap between SNP-CpG significant in mQTL and MPO analyses (based on the 5M imputed SNPs). (b, c) Distribution of heritable CpG and non-heritable CpGs, respectively, and SNP pair in mQTL analysis within 500kb and their corresponding p-values.

A

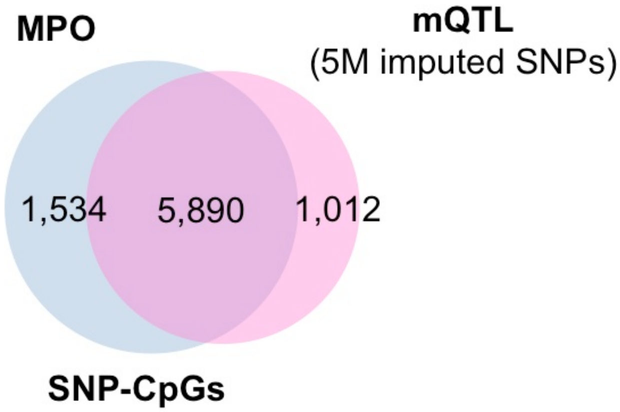

B

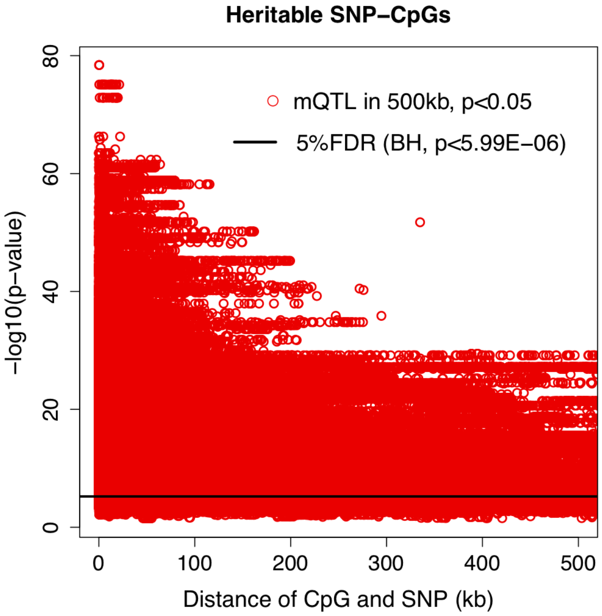

C

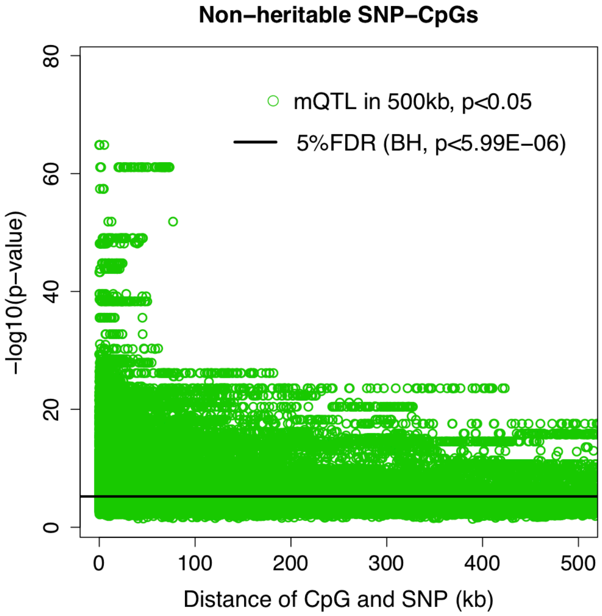

Supplement: File S1 — Figure S1. Hierarchical clustering of high variable CpGs. Figure S2. Manhattan and density plots showing the distribution of associated CpG and SNP pairs across all chromosomes between CpG and SNP pair of 0-2kb (left) and 100kb-1Mb (right) of mQTL analysis using bisREAD SNP data (a, b) and 5M imputed SNP data (c, d), respectively. Distribution of CpG and SNP associations and their corresponding absolute distances of mQTL analysis using bisREAD SNP data (e) and 5M imputed SNP data (f), respectively. Figure S3. Examples of ASM events and regional annotation of CpG associated with ASM. (a, b) Example of allele specific DNA methylation of non-SNP CpG and SNP-CpG, respectively. (b) The presence of T SNP on CpG sites disrupted DNA methylation of that allele. (c, d) Pie charts showing the distribution of non-SNP CpG ASM and SNP-CpG ASM, respectively, in different regions. Figure S4. (a) Venn diagrams showing overlap between SNP-CpG significant in mQTL and MPO analyses (based on the 5M imputed SNPs). (b, c) Distribution of heritable CpG and non- heritable CpGs, respectively, and SNP pair in mQTL analysis within 500kb and their corresponding p-values. (PDF) [file pone.0099313.s014.pdf]
